# Evaluation of headache associated with personal protective equipment during COVID‐19

**DOI:** 10.1002/brb3.2435

**Published:** 2021-11-13

**Authors:** Elham Jafari, Mansoureh Togha, Hossein Kazemizadeh, Samaneh Haghighi, Somayeh Nasergivehchi, Mohammad Saatchi, Shadi Ariyanfar

**Affiliations:** ^1^ Headache Department Iranian Center of Neurological Research Neuroscience Institute Tehran University of Medical Sciences Tehran Iran; ^2^ Advanced Thoracic Research Center Tehran University of Medical Sciences Tehran Iran; ^3^ Department of Geriatric Medicine Ziaeian Hospital Tehran University of Medical Sciences Tehran Iran; ^4^ Department of Neurology Baharloo Hospital Tehran University of Medical Sciences Tehran Iran; ^5^ Research Center in Emergency and Disaster Health University of Social Welfare and Rehabilitation Sciences (USWR) Tehran Iran; ^6^ Department of Clinical Nutrition and Dietetics Faculty of Nutrition and Food Technology Shahid Beheshti University of Medical Sciences Tehran Iran

**Keywords:** headache, mask, protective equipment

## Abstract

**Background:**

The COVID‐19 pandemic has created new conditions for medical staff, forcing them to use personal protective equipment (PPE) for an extended duration of time. Headache is a commonly associated side effect of the use of such equipment among healthcare workers.

**Method:**

In this cross‐sectional study, 243 frontline healthcare workers at four referral hospitals for COVID‐19 were evaluated for the occurrence of headache following the use of PPE and its relationship with blood gas parameters was assessed.

**Results:**

The average age of participants was 36 ± 8 years. Of these, 75% were women. The prevalence of headache after the use of masks was 72.4%, with the N95 mask being the most commonly reported cause of headache (41%). Among patients, 25.1% developed external pressure, 22.2% migraine, and 15.2% tension‐type headaches. Headache was more common in the female gender. Apart from gender, only increased heart rate was significantly associated with headache due to mask use (*p* = .03 and .00, respectively). The mean heart rate was 97.7 ± 13.68 in participants with headache compared to 65.8 ± 35.63 in those without headache. No significant relationship was found between headache and venous blood gas parameters, including oxygen and carbon dioxide partial pressure.

**Conclusion:**

Headache due to PPE is common and can decrease the efficiency of hospital staff performance. Hence, it is necessary to consider this issue among health center personnel and provide modalities to reduce the risk of headache.

## Abstract 2

Given that headache is one of the most common side effects of PPE, we designed a cross‐sectional study to evaluate the occurrence of PPE‐associated headache among the healthcare staff during the COVID‐19 pandemic.

## 1 INTRODUCTION

In December 2019, COVID‐19 was first reported in China and spread rapidly around the world. The World Health Organization announced a pandemic of COVID‐19 in March 2020 (Wang et al., 2020). In Iran, the first diagnosed case of the disease was confirmed on February 20, 2020. By the end of 2020, the total number of diagnosed COVID‐19 patients in Iran reached about 980,000 and the resulting death toll surpassed 55,000.

During this time, frontline healthcare workers experienced major changes in their daily lives. This included wearing specialized masks and clothing, extended hours of stressful work in the hospital, separation from family, along with the fear of contracting the disease and transmitting it to relatives, and the risk of death. WHO recommended that the hospital medical staff should be obliged to wear masks in the normal wards, in addition to gowns and shields in the COVID‐19 wards. This aggravated the fatigue and discomfort of the staff and increased the number of headache complaints associated with the use of these devices (World Health Organization, 2020).

Headache is one of the most commonly associated side effects of the use of personal protective equipment (PPE) among healthcare workers. PPE‐associated headache could be explained by a number of factors, including hypoxia, hypercapnia, local compression on the face or scalp, heat build‐up, and dehydration, as well as anxiety about wearing the device (Headache Classification Committee of the International Headache Society (IHS), 2018; Rebmann et al., 2013). Studies have reported headache in 30% to 80% of personnel who wear the N95‐type mask (Lim et al., 2006; Ong et al., 2020; Radonovich, 2009). The use of protective goggles, shields, and isolation gowns can contribute to the occurrence of headaches (Blau, 2005; Witterseh et al., 2004). Such headaches are more common among those with a history of headache (Ong et al., 2020), but can also occur de novo in those with no history of headache (Ramirez‐Moreno et al., 2020).

Because of the need for PPE use by frontline personnel, especially during the COVID‐19 pandemic, it is necessary to identify and eliminate the factors that lead to intolerance of these devices to help improve individual and group performance of the medical staff. Given that headache is one of the most common side effects of PPE, we designed a cross‐sectional study to evaluate the occurrence of PPE‐associated headache among the healthcare staff during the COVID‐19 pandemic.

## 2 METHODS

This was a cross‐sectional study on medical staff working at four hospitals in Tehran that were among the referral centers for patients with COVID‐19. The subjects were physicians, nurses, and other staff members who worked in the intensive care unit (ICU), emergency, and isolated respiratory wards, as well as in non‐COVID and other paraclinical wards of the hospital who were willing to participate in the study. The study was conducted between April and July 2020, concomitant with the second peak of COVID‐19 in Iran. All participants completed the informed consent form. The study protocol complies with the guidelines of the 2013 version of the Helsinki Declaration and was approved by the Ethics Committee of Tehran University of Medical Sciences.

In accordance with WHO guidelines, the hospital staff were required to wear surgical masks in general wards and N95 masks with or without gowns, shields, and goggles in the COVID‐19 wards. Individuals who had used PPE continuously for at least 4 h were included in the study.

A questionnaire containing the following items was completed by the participants while being supervised by researchers: demographic data, previous history of headache, diagnosis of previous headaches by a neurologist, medical and psychiatric history, drug history, and COVID‐19 status of the individual and/or relatives. Also recorded was the type and duration of use of protective equipment, type of mask (surgical, N95, 3M masks), protective googles, face shields, and isolation gowns. The occurrence of headache after the use of any of this equipment, the characteristics of the headache (quality, location, and duration), and the symptoms associated with the headache (nausea, photophobia, phonophobia, or osmophobia) also were recorded. A de novo PPE‐associated headache was diagnosed in participants without a pre‐existing headache diagnosis who experienced headache closely after PPE usage. We considered an external compression headache for participants who had experienced at least two episodes of headache that occurred within 1 h of PPE usage where pressure was felt primarily at the site of the mask, shield strap, or eyeglass temples that resolved within an hour of decompression.

Blood oxygen saturation and heart rate were recorded by pulse oximeter before and 4 h after the use of PPE or in case of shortness of breath or headache. At the end of 4 h or if shortness of breath or headache occurred, a venous blood sample was taken to measure blood gas parameters, if desired by the individual.

## 3 STATISTICAL METHODS

The continuous variables are expressed as the mean ± standard deviation (SD). The chi‐square test was used to compare proportions for categorical variables. The independent two‐sample *t*‐test and repeated measure test were used for comparison of means. *p*‐values of less than .05 were considered statistically significant. SPSS 18.0 (SPSS Institute; USA) was used for all statistical analyses.

## 4 RESULTS

This study assessed 243 frontline healthcare workers in four hospitals caring for COVID‐19 patients. Table 1 shows the baseline characteristics of these healthcare workers. The average age of participants was 36 ± 8 years. Among them, 75% were women. There was no significant difference between the mean age of the women and men (*p* = .934).

**TABLE 1:** Baseline characteristics of healthcare workers

Characteristics		Healthcare workers (%)	PPE headache group (%)
Gender	Female (%)	82.5	83.2
	Male (%)	17.5	16.8
	Body mass index (mean ± SD)	25.4 ± 5.4	25.3 ± 5.6
Marital status	Single	26.9	29.5
	Married	73.1	70.5
Age	<40 years (%)	68.9	70.2
	≥40 years (%)	31.3	29.8
Pre‐existing primary headache diagnosis		41.3	44.3
Arterial hypertension		1.2	1.7
Previous psychiatric history		7	6.9
Infection of participant or relatives with COVID‐19		17	19.4
Mask usage	Surgical mask	89	91.6
	N95	72	76.6
	N99	3	2.8
	3M	19.7	22.7
	Other masks	10.6	22.7
Goggle usage		48.9	58.2
Face shield usage		48.9	66
Isolation gown usage		54.3	63

The overall prevalence of headache after the use of PPE was 77%. The prevalence of headache after using masks, shields, and goggles was 72.4%, 27.2%, and 27.3%, respectively. Surgical and N95 masks were the most commonly used masks at 89% and 73%, respectively. Other types of masks used included N99, 3M, and other masks. The N95 mask was the most common cause of headache, with about 57% of reports of mask headache occurring in personnel who used this type of mask. The average duration of mask use before the onset of headache was 133.5 ± 113.7 min.

Among patients with PPE headache, 44.3% had a previous history of headache, including migraine and tension types. About 56% of subjects did not report a history of headaches and were considered to have developed de novo PPE‐associated headaches.

Among patients with PPE headache, 25.1% developed external compression, 22.2% migraine, 15.2% tension type, and 37.5% nonspecific headaches. Most PPE headaches were located in frontal parts of the head. The most common headache‐associated symptoms were nausea (37%) and vomiting (14.4%). More females (*p* = .024) than males reported headache occurrence.

An increase in heart rate was significantly associated with headache due to PPE use (*p* = .001). The mean heart rate was 97.7 ± 13.68 in participants reporting headaches, compared to 65.8 ± 35.6 in participants without headaches. Table 2 shows the average pulse rate (PR) at baseline and at 2 to 4 h after mask application. The results of the paired *t*‐test showed that the mean PR increased significantly after 2 to 4 h of mask usage.

**TABLE 2:** Relation between pulse rate and PPE headache

Variable	Mean	SD	Mean difference	*p*‐value^a^
Baseline PR	83.6	10.9	−4.03	≤.001
PR 2–4 h after mask use	87.7	15.2		

^a^ Paired *t*‐test.

Marital status, age, blood pressure, history of headache, duration of PPE use, and history of psychiatric disorders were not significantly related to the occurrence of PPE‐associated headache. The venous blood gas results showed that the mean partial pressure of carbon dioxide (PCO_2_) was higher in participants with PPE headaches (59.4 ± 85.5) compared to participants without PPE headache (46 ± 8.5); however, no statistically significant relationship was found between PPE headache and venous blood gas parameters (Table 3).

**TABLE 3:** Venous blood gas parameters in respondents with and without PPE‐associated headaches

	Headache	*N*	Mean	SD	*p*‐value
pH	Yes	35	7.3	0.02	.558
	No	4	7.3	0.04	
PCO_2_	Yes	35	59.4	85.5	.759
	No	4	46.0	8.5	
HCO_3_	Yes	35	24.1	3.4	.237
	No	4	26.3	2.4	

## 5 DISCUSSION

The COVID‐19 pandemic has forced medical staff to use PPE for an extended duration of time. Mask intolerance has been attributed to diminished visual, vocal, or auditory acuity, excessive humidity or heat, facial pressure, skin irritation, excessive fatigue, and overall discomfort. Flushed face, and pain and pressure on the scalp are among the most common complaints associated with discomfort (Shenal et al., 2012).

Headache is one of the most commonly associated side effects of the use of PPE. The most common cause of headaches in our study was the use of masks (72.4%). The mask most commonly associated with headache was the N95. This is in line with the results of other studies which found a stronger relationship between N95 masks and occurrence of headache (Lim et al., 2006; Ong et al., 2020; Ramirez‐Moreno et al., 2020). In 2003, in a survey of healthcare workers during the severe acute respiratory distress syndrome (SARS) epidemic, about one third reported headaches after wearing the N95 mask (Lim et al., 2006). The strong relationship between headache and N95 mask usage can be attributed to the pressure it exerts on the facial structure, excessive heat, and possibly anxiety about working in a high‐risk environment.

In the literature, the rate of de novo mask‐associated headache has been reported to be 81% with the use of the N95 mask during COVID‐19 (Ong et al., 2020), 26.5% among healthcare providers in Italy (Rapisarda et al., 2021), and 51% in other studies of healthcare workers (Ramirez‐Moreno et al., 2020). The result of our study was the occurrence of de novo PPE‐associated headaches in 55.7% of individuals without a previous history of headaches.

Of the participants with PPE headache, 44.3% reported a previous history of headaches, but there was no significant relation found between the previous history of headache and PPE headache. These results are in contrast to those of a recent study that reported pre‐existing primary headache diagnosis in about one third (29.1%) of respondents and that those participants were more likely to develop de novo PPE‐associated headaches (Ong et al., 2020).

Of the participants with headache in our study, 25.1% developed at least two episodes of external compression headaches that occurred within 1 h of PPE wear, at a maximum at the site of pressure which resolved within an hour of decompression (IHS, 2018). The responsible mechanism in these cases were compression of the trigeminal or occipital nerves branches by the mask, shield strap, or eyeglass temple (Krymchantowski, 2010). There have been no clear reports of external compression headaches due to PPE in the literature.

Some studies have reported prolonged duration of PPE wear as a risk factor for the development of headache (Lim et al., 2006; Ong et al., 2020). The average duration of mask use before the onset of headache in our participants was 133.5 min; however, our results failed to show a significant relationship between PPE headache and the duration of mask use. The female gender was significantly associated with PPE headache in our participants, which is consistent with the findings of other studies (Rapisarda et al., 2021). Other associations mentioned in the literature include a history of asthma, working in emergency units, being a nurse, and a high BMI (Ong et al., 2020; Ramirez‐Moreno et al., 2020; Rebmann et al., 2013). In our study, no relationship was found between PPE headache and BMI or with marital status, age, blood pressure, or history of systemic or psychiatric disorders.

We recorded the blood oxygen saturation and heart rate before and 4 h after the use of PPE or in case of dyspnea or headache. There was a significant relationship between heart rate and headache due to PPE use (*p* = .001). The mean heart rate was 97.7 in participants with headache, compared to 65.8 in those without headache. One study investigating the effects of N95 and surgical facemask use on thermophysiological responses also reported that subjects had significantly lower average heart rates when wearing surgical facemasks than when wearing N95 facemask (Li et al., 2005).

Thirty‐nine participants agreed to perform the venous blood gas test at 4 h after PPE use or when dyspnea or headache occurred. No significant relationship was found between headache and the venous blood gas parameters, including oxygen and carbon dioxide partial pressures or bicarbonate (HCO_3_); however, participants with headache had higher PCO_2_ values. Another study that evaluated the effect of respiratory protective devices on the respiratory function in healthy participants during the COVID‐19 outbreak observed no significant variation in ABG parameters (Ciocan et al., 2020). Other studies that evaluated the effects of long‐term respirator use reported increased CO_2_ levels compared with baseline measures. However, the rise in CO_2_ levels did not reach the clinical definition of hypercapnia and had no toxic effects other than some undesirable symptoms such as fatigue, headache, and loss of concentration (Geiss, 2021; Rebmann et al., 2013).

Wearing an N95 mask during hemodialysis has been significantly associated with reduced oxygen partial pressure and an increased respiratory rate, but this was not confirmed by our study (Kao et al., 2004). Interestingly, one study has reported respiratory alkalosis and hypocarbia after the use of N95 masks and it was quantitatively shown that participant symptoms, including headache, anxiety, tremor, and muscle cramps, were due to respiratory alkalosis and hypocarbia (İpek et al., 2021). Considering all these findings, it appears that PPE usage should not cause significant concern about oxygen delivery to the tissues or carbon dioxide excretion from the lungs. Therefore, healthcare workers can be assured that PPE use for short periods of time is unlikely to have a detrimental effect on their health.

## 6 CONCLUSION

Given the need to continue the use of PPE despite the global onset of vaccinations, it seems necessary to consider measures to reduce their side effects. Headache is one of the most commonly associated complaints of the use of such equipment among healthcare workers and could be explained by local compression on pain sensitive structures, worsening of pre‐existing headaches, or physiological changes in heart rate or respiratory rate, as well as anxiety about wearing the device. Designing new breathing devices that eliminate the risk factors of headache can improve mask tolerance, which can improve PPE compliance and performance among healthcare workers.

## CONFLICT OF INTEREST

The authors declare no potential conflict of interest with respect to the research, authorship, and/or publication of this article.

## AUTHOR CONTRIBUTIONS

Elham Jafari contributed to the study conception, acquisition of data, and drafting of the manuscript. Mansoureh Togha contributed to the study conception and design, analysis and statistics of the data, and the critical revision of the manuscript. Hossein Kazemizadeh, Samaneh Haghighi, and Somayeh Nasergivehchi contributed to acquisition of data. Mohammad Saatchi contributed to analysis and statistics of the data, and Shadi Ariyanfar contributed to data entry and processing.

## FUNDING INFORMATION

The authors have received no funding for this work

### PEER REVIEW

The peer review history for this article is available at https://publons.com/publon/10.1002/brb3.2435

## Data Availability

The datasets used and/or analyzed during the current study are available from the corresponding author on reasonable request.
